# Scale-invariance of receptive field properties in primary visual cortex

**DOI:** 10.1186/1471-2202-8-38

**Published:** 2007-06-11

**Authors:** Tobias Teichert, Thomas Wachtler, Frank Michler, Alexander Gail, Reinhard Eckhorn

**Affiliations:** 1 Department of Physics, NeuroPhysics Group, Philipps University, D-35032 Marburg, Germany; 2 Bernstein Center for Computational Neuroscience (BCCN), German Primate Center, D-37037 Goettingen, Germany

## Abstract

**Background:**

Our visual system enables us to recognize visual objects across a wide range of spatial scales. The neural mechanisms underlying these abilities are still poorly understood. Size- or scale-independent representation of visual objects might be supported by processing in primary visual cortex (V1). Neurons in V1 are selective for spatial frequency and thus represent visual information in specific spatial wavebands. We tested whether different receptive field properties of neurons in V1 scale with preferred spatial wavelength. Specifically, we investigated the size of the area that enhances responses, i.e., the grating summation field, the size of the inhibitory surround, and the distance dependence of signal coupling, i.e., the linking field.

**Results:**

We found that the sizes of both grating summation field and inhibitory surround increase with preferred spatial wavelength. For the summation field this increase, however, is not strictly linear. No evidence was found that size of the linking field depends on preferred spatial wavelength.

**Conclusion:**

Our data show that some receptive field properties are related to preferred spatial wavelength. This speaks in favor of the hypothesis that processing in V1 supports scale-invariant aspects of visual performance. However, not all properties of receptive fields in V1 scale with preferred spatial wavelength. Spatial-wavelength independence of the linking field implies a constant spatial range of signal coupling between neurons with different preferred spatial wavelengths. This might be important for encoding extended broad-band visual features such as edges.

## Background

The primate visual system is capable of processing visual scenes at a large range of different spatial scales. Neurons with receptive fields that are scaled in size with preferred spatial wavelength^1 ^possibly support this achievement. To investigate the potential role of primary visual cortex (V1) in this process we examined to what extent receptive field (RF) properties in V1 scale with preferred spatial wavelength. So far, systematic data for the relation of preferred wavelength and receptive field size are only available for the minimum response field (mRF) [1-6]. However, receptive fields are not described exhaustively by the mRF alone. We investigated scaling properties of several other receptive field measures.

### Scale-independent visual performance

Scale invariance of psychophysical performance has been investigated for a variety of tasks. While detection thresholds of luminance defined gratings depend on scale [7], other tasks such as detection of change in spatial frequency, amplitude or orientation [8,9] of suprathreshold gratings do not depend on spatial scale. Polat and Sagi [10] examined the dependency of lateral interactions on spatial scale in a contrast detection task for human observers. Their results suggest a scale-independent profile of inhibitory and excitatory subregions.

Information in different spatial wavebands is processed by different sets of neurons in V1. Thus, the responses of neurons in V1 are highly dependent on spatial scale. Nevertheless, V1 might support scale-invariant performance as seen in the experiment of Polat and Sagi. This could be achieved by scaled mechanisms implemented in different sets of neurons selective for different spatial wavebands (see [11] for an elaboration of this idea). So far, systematic data are only available for the dependence of the mRF on preferred spatial wavelength [1-6]. The mRF in V1, however, is not a likely candidate to explain the long-range stimulus-stimulus interactions reported by Polat and Sagi. In order to explain their findings we need to understand how different, possibly non-classical, receptive field properties scale with preferred spatial wavelength. In the following we will focus on three measures of receptive field size in V1 that possibly help to understand the mechanisms underlying these long-range stimulus-stimulus interactions.

### Grating summation field (ΣRF) and surround size

Blakemore and Tobin [12] were the first to show that activity of neurons in primary visual cortex to stimuli of a certain orientation can be influenced by oriented stimuli located outside their mRF. These interactions were later examined in more detail [13-22]. The area over which these interactions enhance activity has been termed *grating summation field, contrast summation field *or *spatial summation field*. The size of the grating summation field (ΣRF) is defined as the diameter of the smallest Gabor patch of optimal orientation and spatial wavelength that elicits the strongest response [18,21,23]. Further increase of patch size typically leads to an attenuation of activity. The region with inhibitory influence on neuronal activity has been termed surround [15,20]. While ΣRF size is generally considered to be a less conservative measure of the classical receptive field (cRF), the size of the inhibitory surround is considered to reflect properties of the non-classical receptive field [19,20]. Influences from beyond the cRF are thought to contribute to essential visual tasks like contour integration, masking of a contour by surrounding line-elements and orientation contrast pop-out, among others [24]. So far, most studies investigating properties of the ΣRF and surround size have not varied spatial wavelength (for an exception see [25]). Thus, it is not clear whether the covariance with preferred spatial wavelength observed for the size of the mRF also holds for the ΣRF and the surround size.

### Linking fields (LF)

Coupling of neuronal signals on a millisecond time scale has been suggested to play an important role in the processing of visual information [26-28]. The linking field of a local assembly of neurons has been defined as the area in visual space where appropriate stimuli can initiate synchronized activities with the reference assembly [29]. The linking field has been shown to be larger for neuronal assemblies of like orientation preference [30]. These observations are backed by electrophysiological and anatomical studies [31,32] showing a preference of long-range lateral connections to target areas of like orientation preference. So far, however, it is not known whether the size of the linking field also covaries with the preferred spatial wavelength of the neuronal assemblies.

In contrast to the ΣRF, which is generally considered to be a measure of the cRF [19,20], the linking field is thought to depend on long-range lateral connections [29,33,34] which are known to extend far beyond the cRF [32]. If indeed the two receptive field measures depend on different mechanisms, we would expect these two measures to be independent. Otherwise, we might expect signal coupling to covary with the relative overlap of the respective ΣRFs.

### Spatial wavelength dependence of receptive field measures?

Neurons in V1 represent visual information at specific wavebands centered around their preferred spatial wavelength. To investigate the potential contribution of mechanisms in V1 to scale invariant visual performance, we investigated whether receptive fields of neurons with short preferred spatial wavelength can be considered scaled versions of the receptive fields of neurons with large preferred wavelength. To this aim we measured preferred spatial wavelength, ΣRF and surround size of units in V1. Linking field size was estimated for groups of neurons with short, medium or large preferred spatial wavelength. We first tested the hypothesis that the size of the ΣRF in V1 increases linearly with preferred spatial wavelength. Second, we tested whether the size of the inhibitory surround in V1 increases linearly with preferred spatial wavelength. Third, we tested the hypothesis that the size of the linking field of groups of neurons in V1 scales linearly with preferred spatial wavelength.

## Results

We recorded multiple unit activity (MUA) and local field potentials (LFP) from 77 recording sites in monkey K and 58 recording sites in monkey B. Averaged preferred spatial wavelengths were 0.49 ± 0.47 deg/cyc (3.48 ± 1.99 cyc/deg) for monkey K and 0.21 ± 0.17 deg/cyc (6.74 ± 3.21 cyc/deg) for monkey B (mean ± standard error, see also methods). Despite a wide range of spatial wavelengths presented (~4 octaves), wavelengths of 21 out of 77 recording sites for monkey K and 6 out of 58 for monkey B were only marginally inside or possibly outside of this range. As preferred spatial wavelength of the majority of these recording sites was at the lower range of wavelengths presented, mean preferred spatial wavelength was probably overestimated.

### Preferred wavelength and size of the grating summation field

ΣRF sizes were 0.73° ± 0.33° for monkey K and 0.39° ± 0.16° for monkey B. It is noteworthy that the recordings from different animals and eccentricities (monkey K: 4.1° ± 0.41°, monkey B: 1.97° ± 0.26°) with considerable differences in ΣRF sizes when measured in degrees of visual angle, had similar ΣRF sizes when transformed into millimeters of cortical extent^2 ^(monkey K: 1.73 mm ± 0.8 mm, monkey B: 2.23 mm ± 0.84 mm).

For both monkeys, preferred spatial wavelength and ΣRF size showed a significant positive correlation (two-tailed Spearman rank-correlation test; monkey K: *p *< 10^-9^, *r *= 0.75; monkey B: *p *< 10^-4^, *r *= 0.55, see Fig. 3). For both monkeys identical slopes of 0.41 were found for this relationship. 5% and 95% quantiles of the distribution of slope estimates based on the 1000 preferred stimulus estimates of the bootstrap method (see Methods) were used as confidence interval of the slope estimate. For both monkeys, the confidence intervals did not include zero (monkey K: 0.37 to 0.61, monkey B: 0.18 to 0.55).

**Figure 1:**
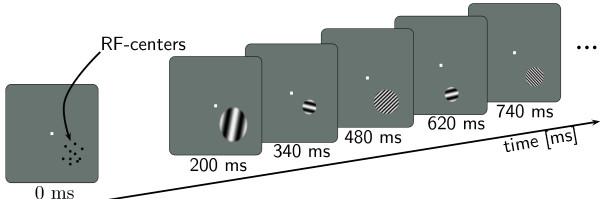
**Stimulation protocol**. While the monkey performed a fixation task, grating patches of 7 different spatial wavelengths and 6 different sizes were presented centered on each cRF. Orientations of stimuli were optimal for each recording site.

**Figure 2:**
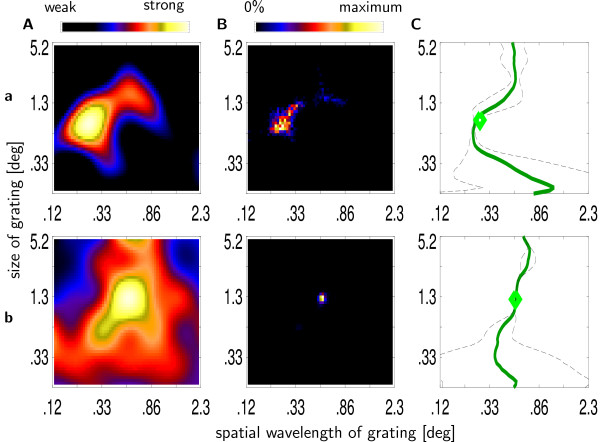
**Mapping of size and wavelength preference**. Determining preferred spatial wavelength and summation field size. Two example recording sites **a **and **b**. (**A**) Interpolated multiple unit activity to stimuli of different sizes and spatial wavelengths. (**B**) Distribution of 1000 global maxima calculated via the bootstrap method. (**C**) Mean (green line) and variance (broken line) of preferred spatial wavelength estimates as a function of stimulus size. The green diamond indicates the mean of the distribution from B.

**Figure 3:**
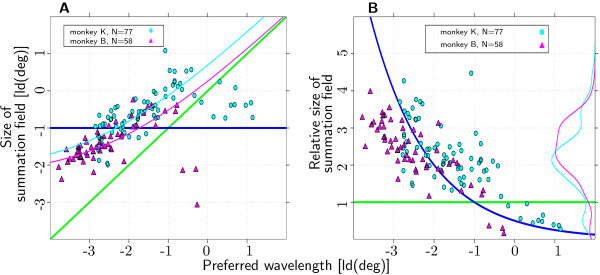
**Preferred Wavelength and ΣRF**. Joint distribution of preferred spatial wavelength and summation field size (135 out of 152 recorded channels; two monkeys, 1 hemisphere each). Dark blue line: model of constant ΣRF size. Green line: model of ΣRF size scaled with preferred spatial wavelength. (**A**) Preferred spatial wavelength and absolute summation field size show a significant positive correlation for both monkeys. Pink and light blue line: fit of the model with additive term as described in Discussion. (**B**) Preferred wavelength and relative summation field size show a significant negative correlation for both monkeys. Pink and light blue line: marginal distributions of relative summation field size.

To test the assumption of linear scaling of ΣRF size with preferred spatial wavelength, we determined relative ΣRF size, defined as ΣRF size divided by preferred spatial wavelength. In the case of linear scaling, relative ΣRF size would be independent of preferred spatial wavelength. From previous reports in the literature [3,6,23], we assume that a relative size of 1 to 1.5 (corresponding to typical simple cell receptive fields with 2 or 3 subunits) can account for spatial frequency bandwidths of the broadly tuned cells in primary visual cortex with bandwidths above 1.2 octaves. In Figure 3A and 3B, the green solid line indicates stimuli with relative sizes of 1. Relative ΣRF sizes were 2.15 ± 0.93 for monkey K and 2.37 ± 0.8 for monkey B (Fig. 3B). The large majority of the recording sites (122 of 135) had relative ΣRF sizes larger than 1.

Relative size of ΣRFs varied systematically with preferred spatial wavelength. In both monkeys there was a significant correlation between preferred spatial wavelength and relative ΣRF (monkey K: *p *< 10^-9^, *r *= 0.67; monkey B: *p *< 10^-9^, *r *= 0.8). Thus, recording sites with short preferred spatial wavelengths had larger relative ΣRF sizes. These results contradict the assumption of linearly scaled ΣRF sizes. Furthermore, the slope of the relation between preferred spatial wavelength and ΣRF size is significantly different from 1, again contradicting a linear relationship.

The distribution of relative ΣRF size is not unimodal (thin pink and light blue line in Fig. 3B). For both animals, a small but clearly separated peak can be observed for the 13 recording sites with relative size less than 1. It is noteworthy that both clusters can be perfectly segregated by preferred spatial wavelength. All recording sites with preferred spatial wavelength larger than ~0.84° (monkey K) and ~0.6° (monkey B), respectively, have relative sizes less than 1. Data from these recording sites were further analyzed in order to decide whether they can be considered a distinct functional subgroup. These analyses (data not shown) suggest that the group of 13 recording sites with relative ΣRF sizes less than 1 may comprise two separate subgroups, one consisting of recordings from multiple units with weak spatial wavelength selectivity, the other consisting of recordings from single neurons.

The fact that for both monkeys the regression lines have slopes below 1 is due to two factors. First, the few recording sites with relative sizes less than one have a pronounced influence on the results. Their properties suggest that these recording sites form a functionally distinct subgroup (see previous paragraph). Second, we observed a systematic decrease in relative ΣRF size with preferred spatial wavelength for the remaining recording sites. The following consideration is proposed to explain the second effect. Preferred spatial wavelength as well as ΣRF size were measured in log_2 _(deg) for all analyses. This has the advantage that any linear function *σ *= *aλ*_*p *_(*σ *= ΣRF size, *a *= scaling factor, *λ*_*p *_= preferred spatial wavelength) has a regression line with slope 1 when plotted on log-log scale. However, affine functions ^3 ^*σ *= *aλ*_*p *_+ *b *with an additive term *b *≠ 0 result in a curvilinear function plotted in log-log scale (red and light blue line in Fig. 3A). These curvilinear functions cannot be described adequately by a typical linear regression analysis. Consequently, the fact that the slope of the regression line is below 1 might merely reflect an affine relation with an additive term unequal to zero.

We slightly modified the standard regression analysis to allow for affine relations between ΣRF size and preferred spatial wavelength of the type *σ *= *aλ*_*p *_+ *b *^4^. For this analysis we excluded recording sites with relative sizes less than 1, as we were interested only in the systematic effect of larger relative ΣRF size for recording sites with short preferred spatial wavelength. Results for both monkeys revealed a scaling factor a in the range between 1 and 1.5 (monkey K, slope a: median:1.4, confidence interval: 0.87 to 1.9, monkey B, median: 1.1, confidence interval: 0.37 to 1.5). The additive term was significantly different from zero for both animals (monkey K, median: 0.22°, confidence interval: 0.12° to 0.36°, monkey B, median: 0.2°, confidence interval: 0.13° to 0.3°). Thus, a purely linear relation between preferred spatial wavelength and ΣRF size could be excluded.

### Dependence of preferred spatial wavelength on stimulus size

**Figure 4:**
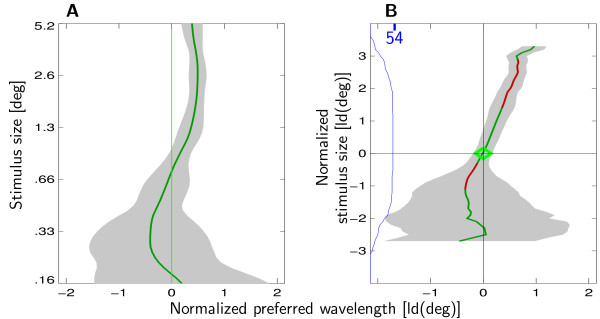
**Preferred Wavelength and stimulus size**. Dependence of preferred spatial wavelength on stimulus size. Results from monkey K (data from monkey B are similar). Green line indicates the preferred spatial frequency estimate at a given stimulus size. The gray area denotes the population mean of the individual variances. **(A) **Normalized preferred spatial wavelength is plotted with respect to absolute stimulus size. Variance of the estimate decreases as stimulus size increases. **(B) **Normalized preferred spatial wavelength is plotted with respect to normalized stimulus size. The blue line indicates the number of recording sites available for averaging at this particular normalized stimulus size. Variance of the estimate decreases as normalized stimulus size approaches 0, i.e., summation field size, but stays constant for larger stimuli. Significant deviations from preferred spatial wavelength are coded in red.

To examine the dependence of preferred spatial wavelength on stimulus size, preferred spatial wavelength estimates were calculated separately for all stimulus sizes^5^. For easy comparison of all recording sites, size-dependent preferred spatial wavelength estimates were subtractively normalized to the size-independent global preferred spatial wavelength estimates. As we were interested in the dependence of preferred spatial wavelength on stimulus size, we excluded recording sites with preferred spatial wavelengths that were only marginally inside the range of wavelengths presented, to avoid floor or ceiling artefacts. Figure 4A shows the influence of stimulus size on normalized preferred spatial wavelength. There is a systematic decrease in the variance of the normalized preferred spatial wavelength estimate (shaded area) with larger stimuli. This is not unexpected as larger stimuli are better localized in the spatial frequency domain.

The effect of increasing stimulus size on response strength depends on ΣRF size. If the stimulus is smaller than the ΣRF, increasing stimulus size leads to stronger responses. If the stimulus is larger than the ΣRF, response strength decreases with increasing stimulus size. Thus, to account for the different ΣRF sizes we subtractively normalized stimulus size to the size of the ΣRF of the recording site in question. A normalized stimulus of size 0 corresponds to a stimulus of ΣRF size. As stimulus size is defined on a logarithmic scale (see Methods), normalized stimulus sizes of -1 and 1 correspond to stimuli of half and twice the size of the ΣRF, respectively.

In Figure 4B normalized preferred spatial wavelength is plotted with respect to normalized stimulus size. Preferred wavelength increases significantly with normalized stimulus size. The dependency is not only significant, but also substantial. When normalized stimulus size is increased from 0 to 2 (corresponding to a 4-fold increase in size), preferred spatial wavelength increases by an average of about half an octave. The data further show that larger normalized stimulus size correlates with a decrease of variance of the preferred spatial wavelength estimate only for normalized stimulus sizes below 0. For normalized stimuli larger than 0, variance of the preferred spatial wavelength estimate is approximately constant. This is especially noteworthy as response strength is maximal for a normalized stimulus size of 0 but decreases for larger stimuli. Still, spatial wavelength bandwidth is not affected by this overall decrease in response strength.

### Preferred wavelength and size of the inhibitory surround

**Figure 5:**
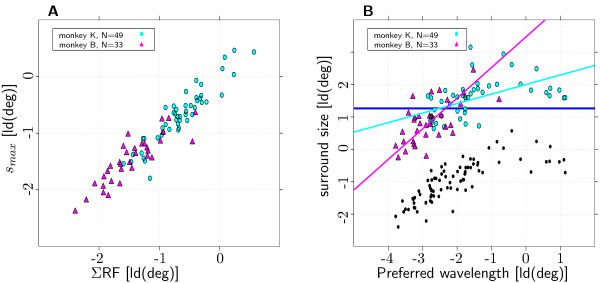
**Preferred Wavelength and surround size**. ΣRF and surround size measures from the DOG method. (**A**) Comparison of ΣRF size estimated with the bootstrap and the DOG method. The two measures are highly correlated (see text for details). (**B**) Joint distribution of preferred spatial wavelength and surround size (82 out of 152 recorded channels; two monkeys, each 1 hemisphere). Dark blue line: model of constant surround size. Preferred spatial wavelength and surround size show a significant positive correlation for both monkeys (see text for details). Pink and light blue line show the regression lines calculated via principal component analysis for monkey K and B, respectively. The black dots show the corresponding ΣRF sizes.

Surround sizes were estimated from a difference of Gaussians (DOG) fit. 49 and 33 recording sites from monkeys K and B, respectively, met the criteria of the DOG fit (see Methods). The DOG fit differs from the bootstrap method used for the estimation of the ΣRF sizes. To compare the two methods, an alternative measure of summation field size (termed *s*_*max*_, to avoid confusion with ΣRF) was extracted from the DOG fit. *s*_*max *_was defined as the patch size yielding the strongest response as estimated by the DOG fit (see Methods). The two measures were highly correlated (monkey K: *p *< 10^-9^, *r *= 0.91, monkey B: *p *< 10^-9^, *r *= 0.89, see Fig. 5A), confirming their validity.

Average surround sizes were 3.22° ± 1.5° for monkey K and 1.96° ± 0.98° for monkey B and thus clearly larger than the ΣRF sizes (see Fig. 5). Surround size was on average 4.88 ± 1.89 times larger than the respective ΣRF for monkey K and 5.1 ± 1.57 times larger for monkey B. This is in keeping with the findings of other groups [21,35].

For each monkey, surround sizes showed considerable variability in the range of 2.5 octaves (see Fig. 5B). A significant fraction of the variance could be explained by preferred spatial wavelength (monkey K: *p *< 10^-5^, *r *= 0.63, monkey B: *p *< 10^-3^, *r *= 0.57). Slopes of the regression lines were 0.29 and 0.95 for monkey K and B, respectively. The fact that the slope was shallower for monkey K than for monkey B was due to the same recording sites that were categorized as outliers in the measurements of the relative ΣRF sizes (see Fig. 5B). While all 3 outliers with relative ΣRF sizes less than 1 for monkey B did not meet the goodness-of-fit criteria for the DOG-fit, all 10 outliers from monkey K did meet the criteria and thus affected the slope estimate. The slope for monkey K increased drastically to 0.94 when the 10 outliers were left out of the sample.

The systematic decrease in relative size with preferred spatial wavelength that was evident for the ΣRF (see Fig. 3) was not present for surround size. Thus, one might speculate that this systematic decrease is unique to the ΣRF. However, it might also be due to a systematic effect of biased sampling possibly imposed by the selection criteria for the DOG-fit. To rule out such an effect, the slope estimates for the dependence of preferred spatial wavelength and ΣRF size were repeated on the subset of recording sites that met the criteria for the DOG-fit. For this subset the slope estimates for the surround were still considerably steeper than those of the ΣRF (0.94 vs. 0.67 for monkey K and 0.95 vs. 0.67 for monkey B). So far only surround sizes for patches of the optimal spatial wavelength, i.e., the wavelength that elicited the strongest responses from the recording site, were considered. To examine the dependency of surround size on stimulus spatial wavelength, surround sizes were measured using patches with spatial wavelengths that were either shorter or longer than the optimal. The results indicated that surround size covaries with the spatial wavelength of the stimulus used for the mapping (data not shown).

### Preferred spatial wavelength and size of the linking field

**Figure 6:**
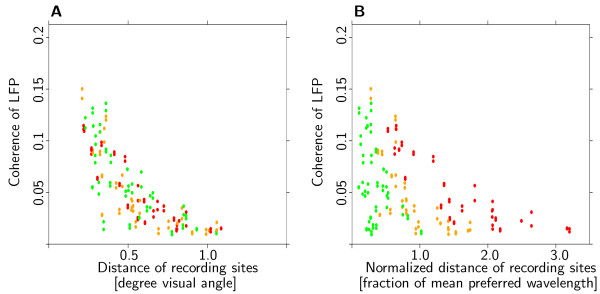
**No systematic influence of preferred spatial wavelength on coupling strength**. Dependence of coupling strength on distance. Pairs of recording sites were separated into three groups with short, medium and long preferred spatial wavelength (red, orange and green dots, see text for details). (**A**) If preferred spatial wavelength were to influence coupling strength, the groups would differ. However, no difference is observed. (**B**) Coupling strength is plotted with respect to distance normalized to mean preferred spatial wavelength. If linking field size were to scale linearly with preferred spatial wavelength, the groups would not differ. However, the three groups are clearly distinct. Results from the other animal and for different coupling measures are comparable. Taken together these results are clear evidence against a scaling of the linking field with preferred spatial wavelength.

As pointed out in Methods, size of the linking field can not be estimated for individual recording sites. Instead, we analyzed differences in the distance dependence of signal coupling strength of all simultaneously recorded pairs. To test for an effect of preferred spatial wavelength on coupling strength, recording sites were divided into groups with short, medium and long^6 ^preferred spatial wavelength. Accordingly, pairs of recording sites were divided into nine groups. The main focus was on pairs for which both recording sites belonged to the same category, i.e., the short-short, medium-medium and long-long spatial wavelength groups. Figure 6A shows dependence of coherence of local field potentials on distance of the recording sites for monkey K. Pairs with short, medium and long preferred spatial wavelength are coded in red, orange and green, respectively. If preferred spatial wavelength were to influence coupling strength, we would expect the groups to be separated. This, however, is obviously not the case. Results for all eight cases, (two monkeys × two signal types × two coupling measures) are similar. To further underline this effect we plotted the same data, this time normalizing distance of the recording sites to mean preferred spatial wavelength (see Fig. 6B). In the case of linear scaling of linking field size with preferred spatial wavelength, we would expect the groups not to differ in this plot. However, the groups show clearly distinct dependence of signal coupling on normalized distance.

The following procedure was used to quantify the results. Several factors, such as distance or difference in orientation preference, have been shown to affect coupling strength. As these factors can not be experimentally manipulated, their influence was accounted for by modeling their effect on coupling strength. The functions used to model signal coupling with distance were chosen individually to capture the properties of the data in question. In 6 out of 8 cases, coupling strength was modeled separately for groups of different relative orientation preferences. Due to a lack of an influence of relative orientation preference on the two LFP coupling measures from monkey B, they were modeled independent of relative orientation preference. Main criterion for the quality of the fit was independence of the residuals from distance. Signal coupling of MUA was modeled using an exponential function (c^i,j=a0e−a1di,j
 MathType@MTEF@5@5@+=feaafiart1ev1aaatCvAUfKttLearuWrP9MDH5MBPbIqV92AaeXatLxBI9gBaebbnrfifHhDYfgasaacH8akY=wiFfYdH8Gipec8Eeeu0xXdbba9frFj0=OqFfea0dXdd9vqai=hGuQ8kuc9pgc9s8qqaq=dirpe0xb9q8qiLsFr0=vr0=vr0dc8meaabaqaciaacaGaaeqabaqabeGadaaakeaacuWGJbWygaqcamaaBaaaleaacqWGPbqAcqGGSaalcqWGQbGAaeqaaOGaeyypa0Jaemyyae2aaSbaaSqaaiabicdaWaqabaGccqWGLbqzdaahaaWcbeqaaiabgkHiTiabdggaHnaaBaaameaacqaIXaqmaeqaaSGaemizaq2aaSbaaWqaaiabdMgaPjabcYcaSiabdQgaQbqabaaaaaaa@3F44@, with c^i,j
 MathType@MTEF@5@5@+=feaafiart1ev1aaatCvAUfKttLearuWrP9MDH5MBPbIqV92AaeXatLxBI9gBaebbnrfifHhDYfgasaacH8akY=wiFfYdH8Gipec8Eeeu0xXdbba9frFj0=OqFfea0dXdd9vqai=hGuQ8kuc9pgc9s8qqaq=dirpe0xb9q8qiLsFr0=vr0=vr0dc8meaabaqaciaacaGaaeqabaqabeGadaaakeaacuWGJbWygaqcamaaBaaaleaacqWGPbqAcqGGSaalcqWGQbGAaeqaaaaa@31CF@: predicted coupling strength and *d*_*i*, *j*_: distance between the two recording sites *i *and *j*). Coupling of LFP was modeled as a linear function of distance and the inverse of distance (c^i,j=a0+a1di,j+a2di,j−1
 MathType@MTEF@5@5@+=feaafiart1ev1aaatCvAUfKttLearuWrP9MDH5MBPbIqV92AaeXatLxBI9gBaebbnrfifHhDYfgasaacH8akY=wiFfYdH8Gipec8Eeeu0xXdbba9frFj0=OqFfea0dXdd9vqai=hGuQ8kuc9pgc9s8qqaq=dirpe0xb9q8qiLsFr0=vr0=vr0dc8meaabaqaciaacaGaaeqabaqabeGadaaakeaacuWGJbWygaqcamaaBaaaleaacqWGPbqAcqGGSaalcqWGQbGAaeqaaOGaeyypa0Jaemyyae2aaSbaaSqaaiabicdaWaqabaGccqGHRaWkcqWGHbqydaWgaaWcbaGaeGymaedabeaakiabdsgaKnaaBaaaleaacqWGPbqAcqGGSaalcqWGQbGAaeqaaOGaey4kaSIaemyyae2aaSbaaSqaaiabikdaYaqabaGccqWGKbazdaqhaaWcbaGaemyAaKMaeiilaWIaemOAaOgabaGaeyOeI0IaeGymaedaaaaa@4808@, with c^i,j
 MathType@MTEF@5@5@+=feaafiart1ev1aaatCvAUfKttLearuWrP9MDH5MBPbIqV92AaeXatLxBI9gBaebbnrfifHhDYfgasaacH8akY=wiFfYdH8Gipec8Eeeu0xXdbba9frFj0=OqFfea0dXdd9vqai=hGuQ8kuc9pgc9s8qqaq=dirpe0xb9q8qiLsFr0=vr0=vr0dc8meaabaqaciaacaGaaeqabaqabeGadaaakeaacuWGJbWygaqcamaaBaaaleaacqWGPbqAcqGGSaalcqWGQbGAaeqaaaaa@31CF@ and *d*_*i*, *j *_as above) as other models using e.g., an exponential function, did not yield residuals that were independent of distance.

For each of the groups, the residuals from the prediction of signal coupling were tested with a non-parametric Wilcoxon test (*α *= .05, not corrected for multiple testing) for significant differences from zero. 9 of the 24 tests (three wavelength groups × two monkeys × two signal types × two coupling measures), indicated residuals significantly different from zero, but no systematic pattern emerged. Thus, the results are consistent with the assumption that in our data the size of the linking field and preferred spatial wavelength are unrelated.

### Different mechanisms for summation field and linking field?

To test whether summation and linking field depend on the same neuronal mechanisms we investigated the correlation between signal coupling and ΣRF overlap. Strength of signal coupling as well as ΣRF overlap depended on the distance of the receptive field centers of the recording sites. Thus, it is not possible to estimate the correlation between overlap and signal coupling in a straightforward way, as the result would reflect the common covariate distance. In other words, the interesting quantity is not the unconditional covariance (Cov [Coupling,Overlap]) but rather the conditional covariance (Cov [Coupling,Overlap|Distance]). Given the sparse sampling of data points, this quantity could not be estimated on a sufficiently fine lattice. Assuming a constant conditional covariance for all values of the condition distance, both variables, signal coupling as well as ΣRF overlap, were modeled as functions of distance. The covariance of the residuals of these fits was interpreted as an estimate of the conditional covariance of ΣRF overlap and signal coupling.

**Figure 7:**
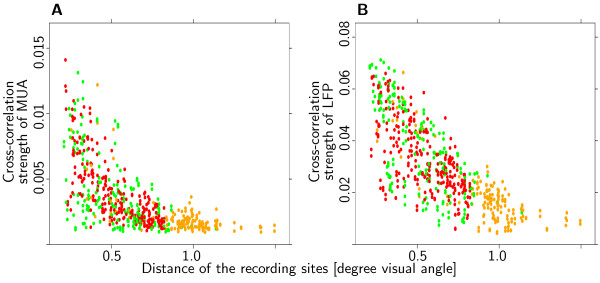
**No systematic effect of overlap on coupling strength**. Two examples that illustrate the lack of a systematic effect of summation field overlap on signal coupling. (**A**) Cross-correlation of MUA, monkey K. (**B**) Cross-correlation of LFP, monkey K. Pairs of recording sites were divided in three groups with small, medium and large residual from the fit of the summation field overlap (red, orange and green dots, see text for details). Signal coupling showed a pronounced dependency on distance of the recording sites. The analysis of residuals revealed a significant negative correlation of overlap and cross-correlation strength of MUA and a positive correlation of overlap and coherence of LFP for monkey K. However, as is evident from the plots, these effects are unsystematic and small. Data from the coherence measure and from the other monkey also fail to reveal a systematic effect of overlap on coupling strength.

The functions used to model signal coupling with distance were the same as described in the previous paragraph. Overlap of ΣRF was more difficult to model. None of the models tested yielded residuals that were completely independent of distance. The dependence of the residuals from distance can be seen in the clustering of pairs with medium-sized residuals in Figure 7 (orange dots). The effect occurs, because pairs of recording sites beyond a certain distance do not have any receptive field overlap at all and are thus easily modeled by functions that predict little or no overlap. The best results were achieved by modeling overlap as an exponential function of distance for monkey K, and as a half-wave rectified linear function for monkey B.

If the amount of overlap influences the strength of signal coupling, we would expect pairs of recording sites with unusually large overlap, i.e., residuals from the overlap estimate larger than zero, to be coupled stronger than pairs of recording sites with unusually low overlap. Figure 7 shows that this is clearly not the case. Pairs with unusually large overlap (green dots) as well as pairs with unusually small overlap (red dots) show identical dependence of signal coupling strength on distance. Note that, although most pairs with little deviation from expected overlap (orange dots) have distances larger than ~.8°, there do exist some pairs with shorter distances. Overall, the distance dependence of all three groups shows hardly any difference.

To back these observations by quantitative analyses, residuals of coupling strength and ΣRF overlap were screened for significant correlations using the non-parametric rank test of Spearman. A total of eight tests were performed (two monkeys × two signal types × two coupling measures). Two of the eight tests indicated a significant positive correlation of the residuals (monkey K: cross-correlation of LFP; monkey B: coherence of LFP). Two of the tests indicated a significant negative correlation (monkey K: cross-correlation and coherence of MUA). All of these correlations explain only very small fractions of the variance of the residuals (up to 12%) and consequently an even smaller fraction of the total variation (up to 6%). Overall, the data do not suggest that ΣRF overlap and coupling strength are related.

## Discussion

Our results show that ΣRF size in V1 increases with preferred spatial wavelength. This increase, however, is not strictly linear. Furthermore, our data suggest that surround size scales linearly with preferred spatial wavelength. In contrast, our results show that the size of the linking field in V1 does not covary with preferred spatial wavelength. Thus, while some properties of receptive fields in V1 scale with preferred spatial wavelength, others clearly do not.

### Preferred spatial wavelength and grating summation field size

We found a substantial variation of ΣRF size with preferred spatial wavelength. The finding that the regression line had a slope above zero but below one excludes both independence of ΣRF size and preferred spatial wavelength as well as linear scaling of ΣRF sizes with preferred spatial wavelength (Fig. 3). Thus, our results are comparable to the data of De Valois et al. [6] and Kulikowski and Vidyasagar [36] for single unit recordings from simple cells. Their results were obtained using stimuli that were well localized in the spatial frequency domain and were thus comparable to the stimuli we used.

The results of the modified regression analysis shows that ΣRF size can be modeled as an affine function of preferred spatial wavelength. The additive term was shown to be in the order of ~0.2° for both monkeys. We discuss three mechanisms that could account for the additive term.

(1) Residual eye movements leading to a misalignment of stimulus and receptive field center could account for the additive term. Mean standard deviation of residual eye-movements on individual fixations were 8.07 and 12.78 minutes of arc in horizontal and vertical direction, respectively. Thus, they are in the order of magnitude to explain the additive term.

(2) The additive term is in the order of magnitude of the jitter of cRF centers at a fixed cortical position [37]. Thus, the additive term might reflect the fact that we recorded MUA from neurons with displaced cRF centers.

(3) As we recorded from supragranular layers and thereby mainly from complex cells, the additive term could be explained by properties unique to complex cells. Studies of complex cells using spike-triggered covariance indicate the presence of spatially shifted subfields in complex cell responses [38]. It is not clear, though, whether the amount of shift dependends on preferred spatial wavelength. If it does not, deviations from linearity corresponding to an additive term could be expected.

Furthermore, we found that preferred spatial wavelength increases with patch size for patches larger than the ΣRF (see Fig. 4B). We suspect that this effect is due to our recording from multiple units. Increasing patch size might have increased the relative contribution of units with slightly longer preferred spatial wavelengths. Thus, this does not imply that scale invariance is implemented in single neurons in a way that preferred spatial wavelength changes with stimulus size.

### Interpretation of summation field size

Receptive fields can be mapped in a variety of ways (mRF, reverse correlation methods, ΣRF, annular minimum response field, surround size). These measures of receptive field size are phenomenological in nature, and their anatomical correlates or their functional implications are not always obvious. Here we propose a functional definition of receptive field size and show that high contrast ΣRF is in accordance with this definition.

Responses of neurons in V1 show properties of localized two-dimensional spatial frequency filters [6,23,39-41], such as selectivity to stimuli at a certain location and of a certain orientation and spatial frequency. To achieve such selectivity in the spatial as well as in the spatial-frequency domain, the input that drives neurons needs to be limited to a certain region in visual space. The size of this area is critical, as neurons would loose their selectivity in the space domain if this region were to be very large. If, in contrast, the region were to be very small, neurons would loose their selectivity in the spatial frequency domain. Spatial frequency selectivity is often mapped using stimuli that are extremely well localized in the spatial frequency domain, such as sinusoidal gratings. If, however, the mapping is conducted with gratings of different sizes, i.e., with varying degrees of localization in the space and spatial frequency domain, it is possible to determine the size that leads to the best selectivity of neuronal responses in the spatial frequency domain. In terms of linear filters, this quantity would be the filter size. We tested two predictions to see if ΣRF size can be considered the equivalent of filter size. First, localization in the frequency domain increases with stimulus size up to ΣRF size. Second, for stimuli larger than the ΣRF size, localization in the spatial frequency domain stays constant.

Figure 4B shows that both criteria are met. Larger stimuli correlate with better localization in the spatial frequency domain only if stimulus size is smaller than the ΣRF. We take this as strong evidence in favor of the hypothesis that ΣRF size reflects the area in visual or cortical coordinates that is used to shape the spatial frequency selectivity. This observation complements the results of Maffei and Fiorentini [13] who reported that the ΣRF contributed to the shaping of the orientation tuning of neurons.

Our results suggest that indeed ΣRF size can be considered an analogon of filter size. As the analogy to linear filter theory is not straightforward we introduce the term *inverse Fourier field *to refer to the area in visual space that is used to enhance spatial frequency and orientation tuning. Using this terminology, our results can be condensed to the observation that, for high contrast gratings, the inverse Fourier field and the grating summation field coincide.

We want to stress again the fact that localization in the frequency domain stays constant although maximum response strength decreases. In a related setting it has been suggested that, probably due to lateral inhibition, information of the intensity of illumination of a large uniform area can be gained only from the borders [42] (see however [43]). Our results indicate that for spatial frequency information of large gratings this is not the case. Spatial frequency information is carried also by neurons with receptive fields centered on a large grating regardless of its size, as long as it is larger than the ΣRF. The effect of decreasing overall response strength while keeping information content constant could be achieved by either enhancing signal-to-noise ratio, possibly by the means of shunting inhibition or by narrowing bandwidth, possibly via subtractive inhibition. Both mechanisms are physiologically plausible and the analyses conducted so far do not favor one over the other. We want to highlight though, that both mechanisms require inhibitory input from neurons with a broad range of preferred spatial wavelengths. A mechanism that only uses input from neurons with similar preferred spatial wavelength is unlikely.

Consequently, these considerations would predict interactions between neurons with different preferred spatial wavelengths. This is in agreement with psychophysical experiments [44,45] which have shown that spatial frequency channels interact at least over a range of two octaves.

### Preferred wavelength and size of the inhibitory surround

Our results show pronounced covariation of preferred spatial wavelength and surround size. When recording sites with relative ΣRF sizes below one are excluded, surround size shows almost linear scaling with preferred spatial wavelength. This seems especially noteworthy, considering that the area outside the ΣRF does not seem to contribute to sharpening of the spatial wavelength tuning (see previous paragraph). However, the fact that surround size covaries with the spatial wavelength of the stimuli indicates that the inhibitory surround is nevertheless mediated by spatial-wavelength selective units.

### Preferred wavelength and linking field size

Our results clearly show that distance dependence of coupling strength does not scale with preferred spatial wavelength (see Fig. 6B). Pairs of recording sites with long preferred spatial wavelength show the same dependence of coupling strength on distance as pairs with short preferred spatial wavelength (see Fig. 6A). Even after ruling out influences from distance and relative orientation preference, no systematic influence of preferred spatial wavelength on coupling strength could be found.  If signal coupling is indeed mediated by lateral long-range connections, our results suggest that the length of these connections does not depend on preferred spatial wavelength of the neurons they connect.

Furthermore, we observed that difference in preferred spatial wavelength does not covary with the strength of signal coupling between two recording sites (data not shown). These results suggest that lateral long-range connections within V1 do not selectively target neurons with similar preferred spatial wavelength. Thus, these observations back the theoretical considerations outlined in the previous section. We suppose that this unspecificity in the wiring of lateral connections does serve a specific purpose. Most stimuli which we encounter in our natural environment have broad frequency spectra. It seems plausible that interactions between neurons with different preferred spatial wavelength play an important role in the coding of natural stimuli.

### Distinct mechanisms for summation and linking field?

Our results indicate different mechanisms for summation and linking field. First, we observed significant signal coupling for non-overlapping ΣRF. This observation extends the finding that signal coupling can be observed when mRFs do not overlap [31]. Second, we found no evidence of a systematic influence of relative ΣRF overlap on signal coupling. Possible mechanisms mediating signal coupling are long-range lateral connections, as discussed in the previous paragraph, or feedback from higher visual areas. Temporal signal coupling in V1 is generally considered to depend on lateral long-range connections [29,33,34]. However, feedback connections from V2 have recently been shown to form orientation-specific connections with V1 [46-48] (but see [49]). Our data regarding the size of the linking field do not favor one possibility over the other. Orientation specificity and spatial spread of lateral as well as feedback connections could explain the observed coupling behavior. However, while linking field size does not depend on preferred spatial wavelength, surround size does. This dissociation suggests different anatomical substrates for these two receptive field properties. Inhibitory influences of the surround can be observed for larger distances than signal coupling. Thus, it is likely that surround size is mediated by feedback connections which cover a larger area than lateral connections [50]. Consequently, lateral connections might be expected to account for the scale-independent linking field.

### Relation of our results to scale-invariant visual performance

In general, our results support the hypothesis that neurons in V1 are involved in scale-independent performance as seen in the detection of change of spatial frequency, amplitude or orientation [8,9] of suprathreshold gratings. Furthermore, our results indicate that the linking field is not the mechanism underlying scale invariant contour integration [51] or lateral facilitation [10,52]. A more likely candidate is the RF surround. First, surround size scales with preferred spatial wavelength. Second, surround sizes are larger and thus better suited to explain the long distances over which facilitation can be observed. The inhibitory effect has been suggested to reduce spontaneous activity and thus improve detection [10].

## Conclusion

Our results show that the widely accepted notion that receptive fields of neurons in V1 are scaled replica of each other (e.g. [53]) is valid in general only to a first approximation. Scaling of receptive field size was found only for the size of the inhibitory surround and, allowing for a nonlinearity in the form of an additive term, also for the grating summation field. We suppose that this coding stage, where information in different spatial wavebands is processed in different sets of neurons with approximately scaled properties, may contribute to scale invariant representations at later stages.

A more complex picture of the scaling properties of receptive fields in V1 emerges when the linking field is taken into account. While information is segregated according to spatial wavelength, there is a certain amount of communication not only within but also between these channels, as indicated by significant cross-correlation and coherence measures. We speculate that this communication is important for the processing of natural stimuli, which are typically broadband. Interestingly, the spatial range of this communication does not depend on preferred spatial wavelength.

Experimental evidence suggests that scaling of receptive field size with preferred spatial wavelength is not found at stages higher in the visual hierarchy [14]. Thus, the spatial-wavelength independence of linking field size in V1 precedes the spatial-wavelength independence of receptive field sizes in higher visual areas. Interestingly, network model studies showed that the range of synchronized activity in one layer may determine the size of receptive fields in the next layer [54]. However, further data would be needed to test whether the spatial-wavelength independence of linking field size in V1 and of receptive field size in higher areas are functionally linked.

Recently, Sceniak et al [18] reported that the size of the ΣRF increases for low-contrast gratings. This increase of ΣRF size seems to be accompanied by a decrease in spatial wavelength tuning width [25]. Thus, it would be of special interest to investigate whether our finding that summation field and inverse Fourier field coincide, is also valid when using low- instead of high-contrast gratings. Furthermore, scaling properties of several other measures of receptive field size such annular minimum response field [20,55] as well as low-contrast summation and linking field, still need to be examined to get the full picture of scaling properties of receptive fields in V1.

## Methods

### Preparation and recordings

Experiments were performed with two male macaque monkeys. Preparation and recording were in accordance with German laws of animal maintenance and experimentation and the guidelines published in the NIH *Guide for the Care and Use of Laboratory Animals*. Extracellular electrical activity was recorded from the upper layers of primary visual cortex, using a 4 × 4 array of singly moveable quartz-isolated platinum-tungsten fiber-microelectrodes [56]. Raw signals (1 Hz – 10 kHz) from each electrode were filtered online to obtain multi-unit activity (MUA: 1–10 kHz band-passed, full-wave rectified, 140 Hz low-passed) and local field potentials (LFP: 250 Hz low-passed). In some cases, single units were isolated using an amplitude window discriminator. During 15 recording sessions (monkey K: 9, monkey B: 6) a total of 240 microelectrode-penetrations were made. On the basis of signal-to-noise ratio during the initial cRF mapping and orientation selectivity, 152 recording sites (monkey K: 90, monkey B: 62) were selected for analysis.

Movements of the left eye were monitored with an infrared camera system (Thomas Recording, Giessen) and sampled at a rate of 250 Hertz. Trials were aborted immediately when the eye-position was outside of the ~0.5° fixation window.

### Minimum response fields (mRF) and orientation preference

Prior to the summation field measurements we determined the receptive field centers and the preferred orientations of all recording sites. Population mRF s were mapped using small bright spots with a Gaussian luminance distribution (*σ *= 4.9 and *σ *= 2.3 minutes of arc, for monkey K and monkey B) flashed for 50 ms at randomly chosen positions on a regular 16 × 16 grid [57]. MUA as a function of spot position was interpolated. Orientation preference of all recording sites was determined by presenting gratings of 8 orientations and 4 wavelengths. The stimuli were presented for 100 ms each, in immediate succession. Stimuli were chosen large enough to simultaneously cover the receptive fields of all recording sites. Preferred orientation for each recording site was determined by inspection of the the peri-stimulus time histograms.

### Stimuli

Summation field (ΣRF) size and preferred spatial wavelength were measured simultaneously using stationary cosine-tapered patches of cosine-gratings. For each of the recording sites, patches of optimally oriented gratings were presented on the respective receptive field centers. The patches varied over 7 different spatial wavelengths and 6 different sizes. In addition, the stimulus set contained a stimulus with zero contrast (*blank patch*). The monkey performed a fixation task while stimuli were presented in pseudo-random sequence for 140 ms each (Fig. 1). Duration of an individual fixation varied between 2.0 and 3.2 s and allowed for the presentation of 14 to 22 stimuli. The first three presentations from each trial and all stimuli that were preceded by a stimulus with identical orientation were discarded because of onset instationarities. For each stimulus, responses to an average of 21 valid presentations were recorded. Spatial wavelengths were logarithmically spaced between 0.11 deg/cyc and 2.17 deg/cyc for monkey K and between 0.07 deg/cyc and 1.33 deg/cyc for monkey B. Stimulus sizes defined as diameter of the patches, ranged between 0.17° and 5.25° for monkey K and 0.10°, and 3.21° for monkey B. Smaller patches and shorter wavelengths were chosen for monkey B to account for different eccentricities of the recording sites (monkey K: 4.1° ± 0.41°; monkey B: 1.97° ± 0.26°).

### Preferred spatial wavelength, summation field size and overlap

For each recording site, the stimulus eliciting the strongest response (preferred stimulus) was estimated by interpolating^7 ^mean responses r¯ij
 MathType@MTEF@5@5@+=feaafiart1ev1aaatCvAUfKttLearuWrP9MDH5MBPbIqV92AaeXatLxBI9gBaebbnrfifHhDYfgasaacH8akY=wiFfYdH8Gipec8Eeeu0xXdbba9frFj0=OqFfea0dXdd9vqai=hGuQ8kuc9pgc9s8qqaq=dirpe0xb9q8qiLsFr0=vr0=vr0dc8meaabaqaciaacaGaaeqabaqabeGadaaakeaacuWGYbGCgaqeamaaBaaaleaacqWGPbqAcqWGQbGAaeqaaaaa@3115@ (1 ≤ *i *≤ 7: spatial wavelength of stimulus, 1 ≤ *j *≤ 6: size of stimulus) to the 7 × 6 different stimuli (Fig. 2A). To obtain an estimate for the variance of the preferred stimulus estimate, the procedure was repeated 1000 times, with noise added to the mean response strengths r˜ijk=r¯ij+nijk
 MathType@MTEF@5@5@+=feaafiart1ev1aaatCvAUfKttLearuWrP9MDH5MBPbIqV92AaeXatLxBI9gBaebbnrfifHhDYfgasaacH8akY=wiFfYdH8Gipec8Eeeu0xXdbba9frFj0=OqFfea0dXdd9vqai=hGuQ8kuc9pgc9s8qqaq=dirpe0xb9q8qiLsFr0=vr0=vr0dc8meaabaqaciaacaGaaeqabaqabeGadaaakeaacuWGYbGCgaacamaaBaaaleaacqWGPbqAcqWGQbGAcqWGRbWAaeqaaOGaeyypa0JafmOCaiNbaebadaWgaaWcbaGaemyAaKMaemOAaOgabeaakiabgUcaRiabd6gaUnaaBaaaleaacqWGPbqAcqWGQbGAcqWGRbWAaeqaaaaa@3E78@ (k: number of repetition). The noise *n*_*ijk *_was independently normally distributed with standard deviation equal to the standard error of response strength for the stimulus in question (*Var*[*n*_*ijk*_] = *Var *[r¯ij
 MathType@MTEF@5@5@+=feaafiart1ev1aaatCvAUfKttLearuWrP9MDH5MBPbIqV92AaeXatLxBI9gBaebbnrfifHhDYfgasaacH8akY=wiFfYdH8Gipec8Eeeu0xXdbba9frFj0=OqFfea0dXdd9vqai=hGuQ8kuc9pgc9s8qqaq=dirpe0xb9q8qiLsFr0=vr0=vr0dc8meaabaqaciaacaGaaeqabaqabeGadaaakeaacuWGYbGCgaqeamaaBaaaleaacqWGPbqAcqWGQbGAaeqaaaaa@3115@]). Figure 2B shows the distribution of these 1000 preferred stimulus estimates for 2 example recording sites. The distribution of preferred stimuli is wider for the recording site in the upper panel than for the recording site in the lower panel, whose preferred stimulus estimates show hardly any variation. ΣRF size and preferred spatial wavelength were defined as size, i.e., the diameter and wavelength ([log_2_(deg)]) of the stimulus representing the mean of the distribution obtained by the bootstrap procedure (green diamond in Figure 2C). Overlap of ΣRFs was defined as the ratio of the area common to both recording sites and the union of the individual areas. The variance of preferred spatial wavelength estimates from the bootstrap method was interpreted as a measure of the localization in the spatial frequency domain. Compared to the standard measure of localization in the spatial frequency domain, this method has the advantage of taking response variability into account. The same method was adapted to obtain estimates of preferred spatial wavelength (green line in Fig. 2C) and spatial frequency selectivity (broken line in Fig. 2C) as a function of stimulus size.

The advantage of using a measure of localization in the spatial frequency domain that takes response strength and variability into account has one drawback. Variability of measured response strength can either be inherent in neuronal responses or can be added by non-neuronal processes, i.e., measurement error. As the method does not distinguish these two potential sources, the measure of localization in the spatial frequency domain may be influenced by both. In cases where the ratio of neuronal to non-neuronal variability is small, our measure of localization in the spatial frequency domain will only reflect a poor signal-to-noise ratio of the recording. To prevent this, we excluded recording sites with a bad signal-to-noise ratio. Signal-to-noise ratio was determined by a time-resolved analysis of variance of the peri-stimulus time histograms. Most recording sites showed a significant increase in variance during the entire stimulus presentation, i.e., over 140 ms. Recording sites with significant increases in variance lasting less than 50 ms were excluded from statistical analyses. Thus, recording sites were excluded that did not show significant differences in response to any high-contrast grating and a zero-contrast stimulus. Finally, recording sites whose variance of the preferred spatial wavelength estimates was considered an outlier from the distribution of these estimates for the whole population, were also excluded from statistical analyses. 77 out of 90 channels from monkey K and 58 out of 62 channels for monkey B met both criteria.

### Surround size

Difference of Gaussian functions (DOG) were fitted to the data for each spatial frequency separately. Response strength *r *was modeled as rf(s)=a(G(sσE)−wIG(sσI))
 MathType@MTEF@5@5@+=feaafiart1ev1aaatCvAUfKttLearuWrP9MDH5MBPbIqV92AaeXatLxBI9gBaebbnrfifHhDYfgasaacH8akY=wiFfYdH8Gipec8Eeeu0xXdbba9frFj0=OqFfea0dXdd9vqai=hGuQ8kuc9pgc9s8qqaq=dirpe0xb9q8qiLsFr0=vr0=vr0dc8meaabaqaciaacaGaaeqabaqabeGadaaakeaacqWGYbGCdaWgaaWcbaGaemOzaygabeaakmaabmaabaGaem4CamhacaGLOaGaayzkaaGaeyypa0Jaemyyae2aaeWaaeaacqWGhbWrdaqadaqaamaalaaabaGaem4CamhabaacciGae83Wdm3aaSbaaSqaaiabdweafbqabaaaaaGccaGLOaGaayzkaaGaeyOeI0Iaem4DaC3aaSbaaSqaaiabdMeajbqabaGccqWGhbWrdaqadaqaamaalaaabaGaem4CamhabaGae83Wdm3aaSbaaSqaaiabdMeajbqabaaaaaGccaGLOaGaayzkaaaacaGLOaGaayzkaaaaaa@488B@ with G(x)=12π∫−x2x2e−x22
 MathType@MTEF@5@5@+=feaafiart1ev1aaatCvAUfKttLearuWrP9MDH5MBPbIqV92AaeXatLxBI9gBaebbnrfifHhDYfgasaacH8akY=wiFfYdH8Gipec8Eeeu0xXdbba9frFj0=OqFfea0dXdd9vqai=hGuQ8kuc9pgc9s8qqaq=dirpe0xb9q8qiLsFr0=vr0=vr0dc8meaabaqaciaacaGaaeqabaqabeGadaaakeaacqWGhbWrdaqadaqaaiabdIha4bGaayjkaiaawMcaaiabg2da9maalaaabaGaeGymaedabaWaaOaaaeaacqaIYaGmiiGacqWFapaCaSqabaaaaOWaa8qmaeaacqWGLbqzdaahaaWcbeqaaiabgkHiTmaalaaabaGaemiEaG3aaWbaaWqabeaacqaIYaGmaaaaleaacqaIYaGmaaaaaaqaaiabgkHiTmaalaaabaGaemiEaGhabaGaeGOmaidaaaqaamaalaaabaGaemiEaGhabaGaeGOmaidaaaqdcqGHRiI8aaaa@43BA@. The free parameter *w*_*i *_corresponds to the strength of the inhibitory surround relative to the excitatory center. Values of 0 and 1 correspond to no and complete supression, respectively. The parameters *σ*_*E *_and *σ*_*I *_reflect the widths of the excitatory center and inhibitory surround mechanisms, respectively. While the *w*_*i*_, *σ*_*E *_and *σ*_*i *_determine the shape of the response profile, the parameter *a *scales absolute response strength. *s*_*max *_was defined as the patchsize *s *that corresponds to the strongest response of the DOG function. When patch size increases towards infinity, estimated response strength *r*_*f *_converges to an asymptotic value *r*_∞ _of *a*(1 - *w*_*I*_). Surround size *s*_*sur *_was defined as the patchsize *s *> *s*_*max *_for which the response strength drops beyond a critical value *c *that was defined as the asymptotic value plus 10 percent of the difference between maximum and the asymptotic value, *c *= *r*_∞ _+ 0.10 (*r*_*f *_(*s*_*max*_) - *r*_∞_). The supression index *C*_Δ _was defined as one minus the fraction between the asymptotic value and the maximal activity (1−r∞rf(smax))
 MathType@MTEF@5@5@+=feaafiart1ev1aaatCvAUfKttLearuWrP9MDH5MBPbIqV92AaeXatLxBI9gBaebbnrfifHhDYfgasaacH8akY=wiFfYdH8Gipec8Eeeu0xXdbba9frFj0=OqFfea0dXdd9vqai=hGuQ8kuc9pgc9s8qqaq=dirpe0xb9q8qiLsFr0=vr0=vr0dc8meaabaqaciaacaGaaeqabaqabeGadaaakeaacqGGOaakcqaIXaqmcqGHsisldaWcaaqaaiabdkhaYnaaBaaaleaacqGHEisPaeqaaaGcbaGaemOCai3aaSbaaSqaaiabdAgaMbqabaGccqGGOaakcqWGZbWCdaWgaaWcbaacbiGae8xBa0Mae8xyaeMae8hEaGhabeaakiabcMcaPaaacqGGPaqkaaa@3DD4@. Criteria for acceptance of the fit were that *s*_*max *_was larger than the smallest stimulus presented, and that the supression index was less than 1. Violations of these criteria indicate that the DOG model did not capture the properties of the data. To exclude sites without inhibitory surround, recording sites with a suppression index less than 0.2 were excluded.

### Linking field

The definition of linking field as the area in visual space where appropriate stimuli can initiate synchronized activities with the reference assembly [29] does not allow for a simple operationalization. The definition implicitly assumes a large number of simultaneously recorded test assemblies as signal coupling depends on a variety of factors such as distance and relative orientation preference. Here we analyzed distance dependence of several coupling measures (coherence and cross-correlation of LFP and MUA) of all simultaneously recorded pairs. Thus, we did not measure linking field size for each individual recording site. Instead we analyzed differences in distance dependence of coupling strength for groups of pairs with different preferred spatial wavelength, thus indirectly estimating the average linking field of neurons of a certain group. Defining a measure of linking field size from the distance dependence of signal coupling is quite arbitrary. As our data revealed no differences between the groups (see Results) we refrained from explicitly choosing one such measure.

A total of 297 and 188 pairs of recording sites from monkey K and monkey B, respectively, were analyzed. For the analysis of signal coupling we considered only responses to stimuli that were centered on the mRF of one of the recording sites in the pair. For each pair, two different analyses were performed, for stimuli centered on each of the two recording sites, respectively. Cross-correlation and coherence were calculated using mean-free MUA and LFP in a Hamming window from 60 to 200 ms after stimulus onset. Taking into account an average response latency of ~60 ms, this interval corresponds to the entire stimulus presentation. A shift predictor [58] was calculated by repeating the same process 100 times with data of the two sites recorded during different, randomly chosen presentations of the stimulus. 5% and 95%-quantiles were used as estimates of lower and upper confidence bounds for the original data. In the case of the cross-correlations, three measures were extracted that were used to define the compound measure of cross-correlation strength used for statistical analysis. Area above the confidence bound (AAC) and area below confidence bound (ABC) were used as an indicator of the amount of cross-correlation strength that could not be explained by stimulus locked components. Cross-correlation strength at zero timeshift (CC_*τ*=0_) was taken as an indicator for correlation versus decorrelation of activity. The compound measure of correlated activity was defined as CC = sign (CC_*τ*=0_) |AAC + ABC|. The mean of the absolute values of this compound measure over all 6 × 7 stimuli was used for statistical analysis. An alternative measure of signal coupling was derived from the data of the coherence measurements. In this case the total area above the upper 95% confidence bound was used as an indicator of signal coupling.

### Statistical analysis

All statistical analyses were performed with the statistics package R version 2.01 [59]. Non-parametric tests (two-sample Wilcoxon test and the Spearman rank-correlation test) were used throughout. As all variables of length^8 ^(cRF size, ΣRF size, and preferred spatial wavelength) were measured in logarithmic units, the statistical tests were performed with data represented on the same logarithmic scale. When reporting mean and standard deviation in e.g. [deg] or [cyc/deg], conversion from the logarithmic to a linear scale was always performed immediately before calculation of mean and standard deviation. All results were rounded to two significant digits.

## Appendix

^1 ^In this paper we use spatial wavelength instead of spatial frequency. We are aware that most studies use spatial frequency, but measuring both receptive field size and spatial scale in identical units, i.e. degrees of visual angle, allows for a more intuitive presentation of the results.

^2 ^Cortical magnification factor *M *was calculated via the formula from van Essen [60]*M *= 13 *E*^1.22^, *E *= eccentricity.

^3 ^Functions of the form *σ *= *aλ*_*p *_+ *b *are generally considered to be linear, but in the strict mathematical sense they are affine functions. The additive term is a non-linearity. Matters are further complicated by the fact that a linear regression generally fits an affine, and not a linear function to the data, unless the additive term is explicitly set to zero.

^4 ^This method is not identical to performing a linear regression on the linearly scaled values, as the error terms are still on a logarithmic scale. This, however, is the only difference.

^5 ^To achieve fine resolution we used interpolated data, see methods.

^6 ^Cutoff values were chosen to achieve a comparable number of recording sites in each group: monkey K: short < -2; long > -1.1 (wavelength in log_2_), monkey B: short < -3.08; long > -2.47

^7 ^data were interpolated using radial basis functions with an inverse multiquadratic kernel and re-sampled on a 61 × 51 grid.

^8 ^Only distance between cRF centers was measured in units of degree of visual angle.

## Authors' contributions

TT participated in the design and realization of the research, planned and performed the data analysis and participated in the writing of the manuscript. TW participated in the design and realization of the research and the writing of the manuscript. FM participated in the writing of the stimulation software. AG participated in the design of the research and in the writing of the stimulation software. RE participated in the design of the research and the writing of the manuscript.
